# Expression and localisation of two-pore domain (K2P) background leak potassium ion channels in the mouse retina

**DOI:** 10.1038/srep46085

**Published:** 2017-04-26

**Authors:** Steven Hughes, Russell G. Foster, Stuart N. Peirson, Mark W. Hankins

**Affiliations:** 1 The Nuffield Laboratory of Ophthalmology, Sleep and Circadian Neuroscience Institute, Nuffield Department of Clinical Neurosciences, University of Oxford, Sir William Dunn School of Pathology, OMPI G, South Parks Road, Oxford, OX1 3RE, UK

## Abstract

Two-pore domain (K2P) potassium channels perform essential roles in neuronal function. These channels produce background leak type potassium currents that act to regulate resting membrane potential and levels of cellular excitability. 15 different K2P channels have been identified in mammals and these channels perform important roles in a wide number of physiological systems. However, to date there is only limited data available concerning the expression and role of K2P channels in the retina. In this study we conduct the first comprehensive study of K2P channel expression in the retina. Our data show that K2P channels are widely expressed in the mouse retina, with variations in expression detected at different times of day and throughout postnatal development. The highest levels of K2P channel expression are observed for Müller cells (TWIK-1, TASK-3, TRAAK, and TREK-2) and retinal ganglion cells (TASK-1, TREK-1, TWIK-1, TWIK-2 and TWIK-3). These data offer new insight into the channels that regulate the resting membrane potential and electrical activity of retinal cells, and suggests that K2P channels are well placed to act as central regulators of visual signalling pathways. The prominent role of K2P channels in neuroprotection offers novel avenues of research into the treatment of common retinal diseases.

The retina is the primary light detecting tissue in mammals and performs an essential role in vision. The retina is organised into three cellular layers containing six major neuronal cell types, each tasked with performing different functional roles^1^,^2^. Rods and cones of the outer retina detect light, and are modulated by feedback inhibition from horizontal cells. Bipolar cells and amacrine cells of the inner retina modulate and integrate rod and cone driven signals and provide synaptic input to retinal ganglion cells (RGCs) that ultimately project light information to retinorecipient areas of the brain. Müller cells are the major glial cell type of the retina and provide homeostatic and metabolic support for retinal neurons, and may also perform roles in transmission of light to outer retinal photoreceptors^3^,^4^. Like all neuronal tissues, the cells of the retina use changes in membrane potential and intracellular ion concentrations to generate and transmit electrical signals, and ultimately encode visual information. However, the signalling pathways of the retina are highly complex and the precise roles performed by distinct classes of ion channels are currently unknown. Most notably, the K^+^ ion channels responsible for regulating the resting membrane potential and cellular excitability of retinal ganglion cells (RGCs) remain to be determined. RGCs are the first cells in the visual pathway to encode light as tonic and transient patterns of action potential firing^5^ and are required to maintain responses over a wide range of light intensities^6^. The regulation of RGC excitability is therefore fundamental to preventing signal saturation and increasing the dynamic range of the retina. Furthermore, different components of the visual signal are encoded by distinct subtypes of RGC which show characteristically different levels of sustained activity and responses to light (sustained and transient ON, OFF and ON-OFF responses for example)^2^,^7^. However, the mechanisms that set resting membrane potential and regulate these differing levels of tonic and transient activity in RGCs remain to be fully determined^8^,^9^.

The importance of K^+^ leak currents to resting membrane potential and neuronal function was first proposed in the original Hodgkin-Huxley model of action potential generation^10^. The continuous efflux of K^+^ ions across the cell membrane is essential for setting a hyperpolarised resting cell membrane potential and directly influences the likelihood, duration and frequency of action potential firings^11^,^12^. However, it was not until the late 1990’s, and the discovery of the two-pore domain (K2P) potassium channels^13^,^14^, that the ion channels responsible for generating background leak K^+^ currents were identified. K2P channels are characterised by the presence of two pore forming regions and four trans-membrane spanning (4TMS) regions in each channel subunit and unlike other classes of K^+^ channels form functional dimers (not tetramers). Typically these channels elicit spontaneously active, outwardly rectifying ‘background leak’ type K^+^ conductances and show no classical time-dependent or voltage-dependent activity^11^,^15^,^16^,^17^,^18^,^19^.

To date at least 15 different K2P channels have been identified in mammals, that can be broadly split into six subfamilies according to their biophysical characteristics and pharmacological properties^11^,^15^,^17^,^18^,^20^. These include the weak inward rectifiers (TWIK-1, TWIK-2 and TWIK-3), acid sensitive rectifiers (TASK-1, TASK-3, and TASK-5), lipid sensitive mechano-gated channels (TREK-1, TREK-2 and TRAAK), halothane inhibited channels (THIK-1 and THIK-2), alkaline sensitive channels (TALK-I, TALK-2 and TASK-2) and the fatty acid inhibited calcium activated channel (TRESK). In addition to important biophysical stimuli, such as pH, temperature and mechanical pressure, K2P channels are also targets for a range of clinically important drugs including inhalational and local anaesthetics, anti-psychotics, anti-depressants and neuroprotective agents (for reviews see refs 19, 21, 22, 23, 24, 25). K2P channels are also widely modulated by G protein signalling pathways and show sensitivities to a diverse array of second messenger systems^11^,^15^,^17^,^18^,^26^,^27^. K2P channels are therefore capable of fine-tuning levels of cellular excitability under a wide range of biochemical and physiological conditions, and can be considered as central regulators of neuronal activity. Alternate splicing^28^,^29^,^30^,^31^, alternative translation initiation^32^, and hetero-dimerisation of K2P channel subunits^33^,^34^,^35^ further increases the level of functional complexity achievable by K2P channels.

At least one member of the K2P channel family has been identified in all mammalian cells investigated (neuronal and non-neuronal), with numerous cell types shown to express multiple K2P channels simultaneously (for example see ref. 36). K2P channels have been assigned prominent roles in many physiological systems, including cardiovascular^37^,^38^, pain^39^,^40^, respiration^41^, hearing^42^,^43^, taste^44^, anaesthesia^22^,^45^,^46^ and sleep^47^,^48^,^49^. K2P channels have also been implicated in a number of pathological conditions, including autoimmune and degenerative diseases^50^, tumourgenesis^51^,^52^, mental retardation (Barel-Birk-syndrome)^53^, migraine^54^, ischemia^46^,^55^,^56^, epilepsy^46^ and depression^46^,^57^,^58^. However, despite the accepted importance of K2P channels to neuronal function^12^,^16^,^17^,^59^, the expression and role of these channels in the retina remains largely unexplored. To date only one study has confirmed a role for K2P channels in the retina. Ford *et al*.^60^, have shown that TREK-1 contributes to slow after-hyperpolarisation events and regulates the frequency of retinal waves within starburst amacrine cells during early postnatal development. However, this study did not investigate the role of TREK-1 (or other K2P channels) in the adult retina.

In this study we have used qPCR and immunohistochemistry to conduct the first comprehensive study of K2P channel expression in the mouse retina. We show that K2P channels are widely expressed in the retina, with multiple K2P channels detected within Müller cells and retinal ganglion cells. These data offer new insight into the mechanisms that regulate electrical activity within the retina and indicate that K2P channels likely perform important physiological functions within visual signalling pathways.

## Results

### qPCR profiling of K2P channel mRNA expression in the mouse retina

**Figure 1:**
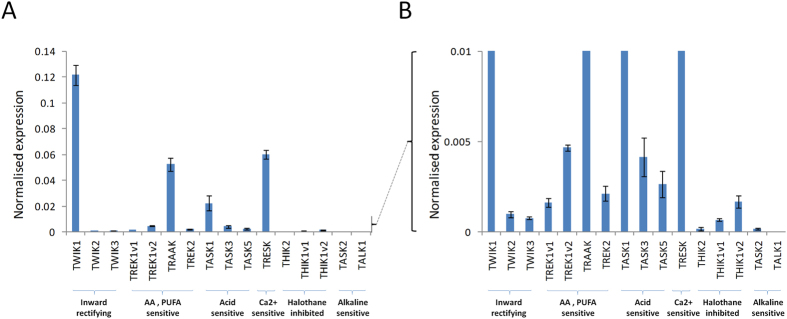
qPCR analysis of K2P channel expression in the adult mouse retina. (**A**) qPCR analysis shows expression of multiple K2P channel mRNA transcripts in the wildtype mouse retina (C3H, non *rd*) (n = 5, P130–135, retinae collected at Zeitgeber time ZT8, 8 hours after light onset). (**B**) The same graph shown in **(A)** with an enlarged scale to highlight the expression of K2P channels expressed at lower levels. Data are shown following normalization to the expression of three house-keeping genes. All data are shown as mean ± S.E.M.

Quantitative PCR analysis shows widespread expression of K2P channel mRNA in the wildtype mouse retina (n = 5, P135, tissue collected at Zeitgeber time ZT8, 8 hours after light onset) (Fig. 1). The highest levels of mRNA expression were detected for TWIK-1, TASK-1, TRAAK, and TRESK, with mRNA transcripts for TWIK-2, TWIK-3, TREK-1, TREK-2, TASK-3, TASK-5 and THIK-1 detected at lower levels. mRNA encoding TASK-2, THIK-2 and TALK-1 were not detected in retina cDNA samples (as determined by a lack of measurable CT value after 40 cycles of PCR). Levels of K2P channel mRNA expression are seemingly highly dynamic in the mouse retina with developmental and circadian variation of expression detected for nearly all channels investigated (Supplementary Figure 1). For the majority of K2P channels, including TWIK-2, TREK-1, TRAAK, TASK-1, TASK-3, TASK-5, and TRESK, a clear up regulation of mRNA expression was detected throughout postnatal development with expression typically reaching maximal levels by P14. By contrast, TWIK-1 and TASK-2 were expressed at higher levels early during postnatal development (all tissue collected at ZT8) (Supplementary Figure 1A). Furthermore, numerous K2P channels were found to exhibit rhythmic patterns of mRNA expression under 12:12 light dark cycles (Supplementary Figure 1B). For the majority of channels, including TWIK-1, TWIK-2, TRAAK, TASK-1, TASK-3 and TASK-5, levels of mRNA expression were highest during the subjective night and typically lowest 8 hours after light onset (ZT8). In contrast, TREK-1 and TRESK showed opposing patterns of expression, with mRNA for these channels peaking at ZT8.

### Localisation of K2P channel proteins in the mouse retina

**Figure 2:**
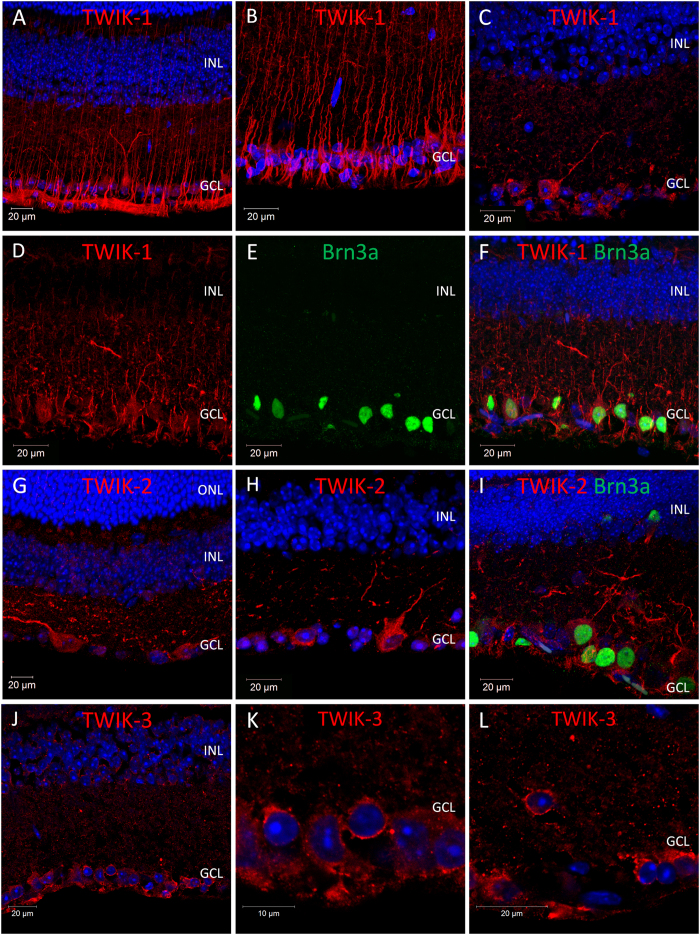
Expression and localisation of inward rectifying TWIK-type K2P channels in the mouse retina. (**A**–**C**) Images showing the localisation of TWIK-1 immunoreactivity in the mouse retina. (**D**–**F**) Co-localisation of TWIK-1 and the RGC marker Brn3a. (**G**,**H**) Images showing the localisation of TWIK-2 immunoreactivity in the mouse retina. (**I**) Co-localisation of TWIK-2 and Brn3a. (**J**–**L**) Images showing the localisation of TWIK-3 immunoreactivity in the mouse retina. ONL; outer nuclear layer, INL; inner nuclear layer, GCL; ganglion cell layer, Brn3a; brain-specific homeobox/POU domain protein 3A, DAPI nuclear counterstain is shown in blue.

**Figure 3:**
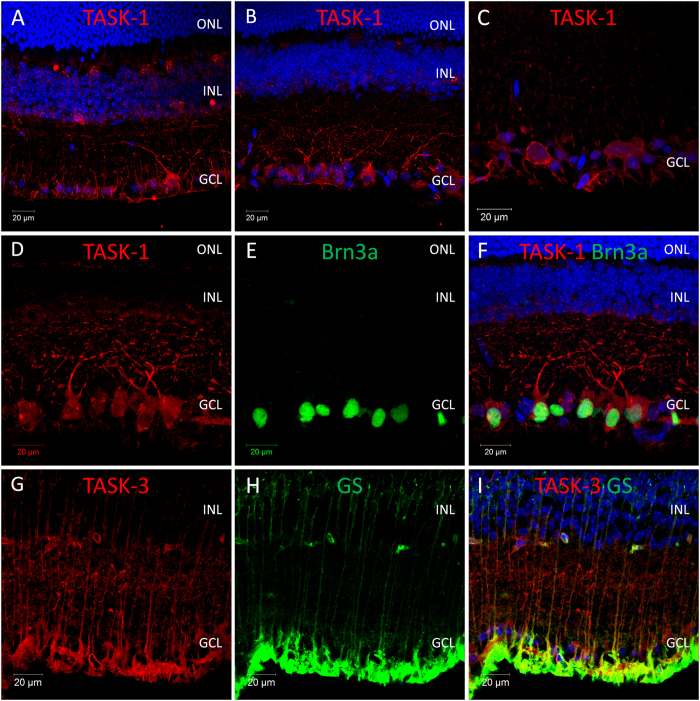
Expression and localisation of acid sensitive TASK-type K2P channels in the mouse retina. (**A**–**C**) Images showing the localisation of TASK-1 immunoreactivity in the mouse retina. (**D**–**F**) Co-localisation of TASK-1 and the RGC marker Brn3a. (**G**–**I**) Images showing the localisation of TASK-3 immunoreactivity in the mouse retina and co-localisation with the Müller cell marker glutamine synthetase (GS). ONL; outer nuclear layer, INL; inner nuclear layer, GCL; ganglion cell layer, Brn3a; brain-specific homeobox/POU domain protein 3A, GS; glutamine synthetase, DAPI nuclear counterstain is shown in blue.

**Figure 4:**
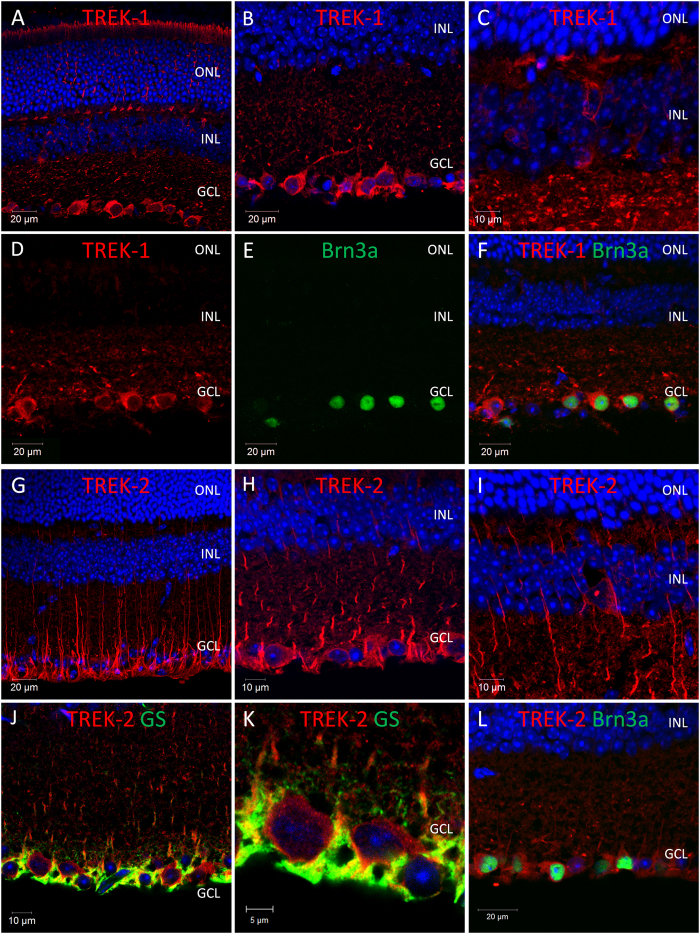
Expression and localisation of arachidonic acid mechanosensitive TREK- type K2P channels in the mouse retina. (**A**–**C**) Images showing the localisation of TREK-1 immunoreactivity in the mouse retina. (**D**–**F**) Co-localisation of TREK-1 and the RGC marker Brn3a. (**G**–**I**) Images showing the localisation of TREK-2 immunoreactivity in the mouse retina. (**J**,**K**) Co-localisation of TREK-2 and the Müller cell marker glutamine synthetase (GS). (**L**) Co-localisation of TREK-2 and Brn3a. PR; photoreceptors, ONL; outer nuclear layer, INL; inner nuclear layer, GCL; ganglion cell layer, Brn3a; brain-specific homeobox/POU domain protein 3A, GS; glutamine synthetase, DAPI nuclear counterstain is shown in blue.

**Figure 5:**
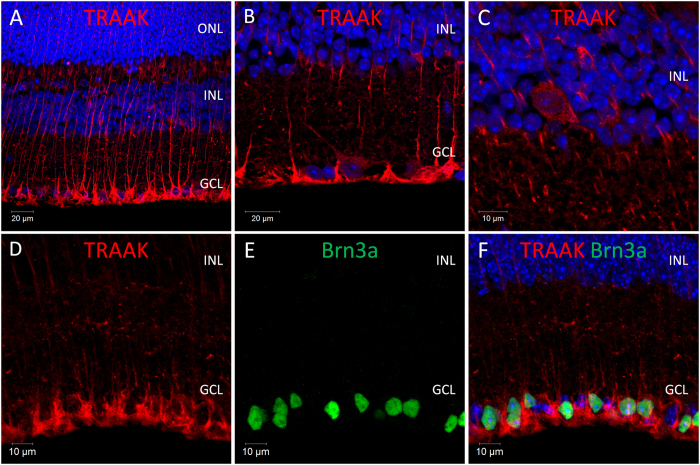
Expression and localisation of arachidonic acid mechanosensitive TRAAK channel in the mouse retina. (**A**–**C**) Images showing the localisation of TRAAK immunoreactivity in the mouse retina. (**D**–**F**) Co-localisation of TRAAK and the RGC marker Brn3a (brain-specific homeobox/POU domain protein 3A). ONL; outer nuclear layer, INL; inner nuclear layer, GCL; ganglion cell layer. DAPI nuclear counterstain is shown in blue.

**Figure 6:**
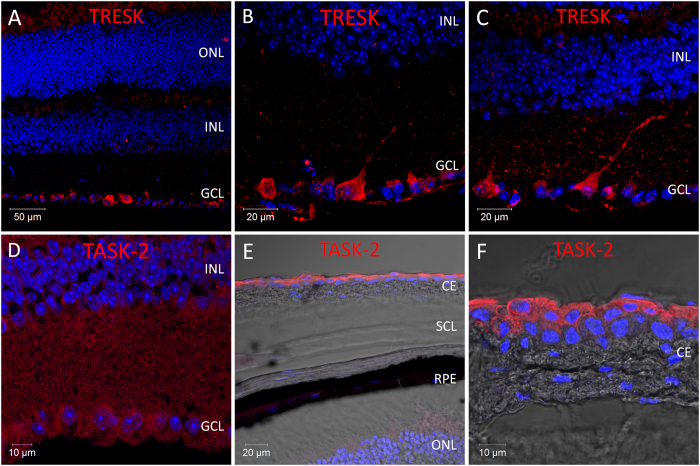
Expression and localisation of calcium sensitive TRESK channel and the alkaline sensitive TALK-type K2P channel TASK-2 in the mouse retina. (**A**–**C**) Images showing the localisation of TRESK immunoreactivity in the mouse retina. (**D**,**E**) Images showing the localisation of TASK-2 immunoreactivity in the mouse retina. CE; conjunctival epithelium, SCL; sclera, RPE; retinal pigment epithelium, ONL; outer nuclear layer, INL; inner nuclear layer, GCL; ganglion cell layer, DAPI nuclear counterstain is shown in blue.

Following qPCR analysis, immunohistochemistry was performed with anti-K2P channel antibodies to confirm the expression and localisation of K2P channel proteins within the mouse retina. Overall our data show that TWIK-type, TREK-type and TASK-type channels are widely expressed in the mouse retina, with the highest levels of expression detected within retinal ganglion cells (TASK-1, TREK-1, TWIK-1, TWIK-2, TWIK-3, and to a lesser extent TREK-2, TRAAK, and TRESK) and Müller cells (TWIK-1, TASK-3, TRAAK, and TREK-2) (Figs 2, 3, 4, 5, 6, results summarised in Table 1).

### Localisation of weak inward rectifying TWIK type K2P channels in the mouse retina

**TWIK-1**; Expression of TWIK-1 was detected in numerous cell types of the adult mouse retina (Fig. 2), and showed a different pattern of expression throughout postnatal development (Supplementary Figure 2). In the adult retina, TWIK-1 immunoreactivity was detected predominantly in Mϋller cells and a subset of cells located in the ganglion cell layer (GCL) (Fig. 2A–C). The intensity of TWIK-1 immunoreactivity within Müller cells was found to be somewhat variable across the adult retina, with areas of high and low expression often observed in the same retina sections. Based on our analysis it was not possible to determine the extent or patterning of this distribution across the entire retina. Double labelling for the retinal ganglion cell marker Brn3a (brain-specific homeobox/POU domain protein 3A) that is expressed in the majority, but not all classes of retinal ganglion cells^61^, confirms the expression of TWIK-1 within 30.7% of RGCs (68 of 196 cells counted), but was also detected in a number of Brn3a negative cells (Fig. 2D–F). In addition to expression of TWIK-1 in Mϋller cells and RGCs of the adult retina, we also observed TWIK-1 immunoreactivity during early development in a population of cells located in the middle of the neuroblastic cell layer, resembling developing horizontal cells (Supplementary Figure 2). Immunoreactivity was weak in these cells at P3, and was increased by P5. By P10 the morphology of these cells clearly resembled horizontal cells, with strong labelling of processes evident in the forming outer plexiform layer. TWIK-1 immunoreactivity was absent from these presumed horizontal cells after P10. Although expression of the RGC marker Brn3a was detected as early as P0, TWIK-1 immunoreactivity was absent from these developing ganglion cells prior to P10 and shows a significant increase by P14 and P30 (Supplementary Figure 2). TWIK-1 immunoreactivity was absent from Müller cells during early postnatal development (Supplementary Figure 2).

**TWIK-2**; TWIK-2 immunoreactivity was consistently detected in cells of the GCL (Fig. 2G,H). Double labelling with Brn3a confirms these cells to be RGCs, with TWIK-2 immunoreactivity detected for 60.8% (73 of 120 cells counted) of all Brn3a positive RGCs in the adult retina including RGCs with multiple distinct morphologies (Fig. 2I). Typically, the highest level of TWIK-2 immunoreactivity was observed along dendrites of immunoreactive cells, with lower levels of immunoreactivity observed for cell bodies. Consistent with qPCR analysis (Supplementary Figure 1), levels of TWIK-2 expression are low during early postnatal development with TWIK-2 immunoreactivity not detected within RGCs prior to P10, and increased by P14 and P30 (Supplementary Figure 3). **TWIK-3**; TWIK-3 immunoreactivity was observed for 50.5% (54 of 107 cells counted) of cells within the ganglion cell layer of the adult retina (Fig. 2J–L). TWIK-3 immunoreactivity was largely restricted to the cell membrane of reactive cell bodies, with only minimal labelling detected along cellular process. However, due to species limitations it was not possible perform double labelling with this TWIK-3 antibody and the Brn3a antibody used to identify RGCs (both raised in goat).

### Localisation of acid sensitive TASK type K2P channels in the mouse retina

**TASK-1**; Of all the K2P channel antibodies tested, the highest levels of immunoreactivity were observed for TASK-1 (Fig. 3). In adult retina TASK-1 immunoreactivity was restricted to the inner retina, with the highest levels of staining detected for cells located in the GCL (Fig. 3A–C). Double labelling with Brn3a and TASK-1 antibodies confirmed the expression of TASK-1 in 67.4% of RGCs (91 of 135 cells counted) with high levels of TASK-1 immunoreactivity detected on the cell bodies and also along the dendrites of labelled cells (Fig. 3D–F). Lower levels of TASK-1 staining were also observed for a subset of cells located in the INL, consistent with the location of amacrine cells (Fig. 3A). TASK-1 immunoreactivity was detected throughout postnatal development. Consistent with our qPCR data, low levels of TASK-1 immunoreactivity were observed for Brn3a positive RGCs as early as P0, with levels of TASK-1 staining increasing through postnatal development and reaching maximal levels by P14 (Supplementary Figure 4). In addition, during postnatal development TASK-1 immunoreactivity was also observed for cells located in the neuroblastic cell layer, with morphologies resembling developing horizontal cells. TASK-1 immunoreactivity was evident in these cells only between P3 and P10, was absent from these cells by P14, and was not detected in P30 adult samples (Supplementary Figure 4).

**TASK-3**; TASK-3 immunoreactivity was predominantly observed for Müller cells (Fig. 3G), where expression of TASK-3 within Müller cells was confirmed by double labelling for the Mϋller cell marker glutamine synthetase^62^ (Fig. 3G–I)**. TASK-5**; Due to the low levels of TASK-5 mRNA detected in the mouse retina we have not investigated the expression and distribution of TASK-5 protein.

### Localisation of arachidonic acid and mechanosensitive sensitive TREK/TRAAK type K2P channels in the mouse retina

**TREK-1**; TREK-1 immunoreactivity was detected for multiple cell types in the adult mouse retina (Fig. 4). The highest levels of TREK-1 immunoreactivity were observed for cells located in the GCL (Fig. 4A,B), with 63.6% (75 of 118 cells counted) of Brn3a positive RGCs labelled for TREK-1 (Fig. 4D–F). Weaker labelling of TREK-1 was also detected for a number of cells located on the inner surface of the INL, likely amacrine cells (Fig. 4A), and a number of cells positioned towards the outer surface of the INL with morphologies resembling horizontal cells (Fig. 4C). TREK-1 immunoreactivity was also detected in the outer retina, with TREK-1 labelling observed for photoreceptor outer segments and also photoreceptor pedicles located in the outer plexiform layer (OPL) (Fig. 4A). Consistent with qPCR analysis (Supplementary Figure 1), expression of TREK-1 protein was not detected in the retina prior to P10 (Supplementary Figure 5).

**TREK-2**; TREK-2 immunoreactivity was detected predominantly within Mϋller cells (Fig. 4G,H and J,K). However, low levels of TREK-2 immunoreactivity were also observed for a number of cells located in the GCL (Fig. 4H and K) and also the INL (typically only 3–5 cells per retina section) (Fig. 4J), including 30.3% (30 of 99 cells counted) of Brn3a positive RGCs (Fig. 4L). **TRAAK**; The pattern of TRAAK immunoreactivity observed in the adult mouse retina was highly similar to that observed for TREK-2 (Fig. 5). TRAAK immunoreactivity was detected at high levels within Mϋller cells (Fig. 5A,B), with lower levels of staining observed for a subset of cells located in the GCL (Fig. 5B) and INL (Fig. 5C). Low levels of TRAAK immunoreactivity were detected for 15.5% (14 of 90 cells counted) of Brn3a positive RGCs. (Fig. 5D–F). Consistent with our qPCR data, TRAAK immunoreactivity was not observed in the retina prior to P14.

### Localisation of calcium sensitive TRESK, alkaline sensitive TALK type and halothane inhibited THIK type K2P channels in the mouse retina

**TRESK**; Despite the high levels of TRESK mRNA detected by qPCR analysis, the levels of TRESK immunoreactivity detected in the adult mouse retina were low with the antibody used in this study (Fig. 6A–C). TRESK immunoreactivity was limited to a small number of cells in the GCL, typically only 4–6 cells per section. However, positive staining with this antibody was inconsistent between different retina samples.

**TASK-2**; Our qPCR analysis indicates only minimal expression of any TALK type channels in the adult mouse retina. Consistent with our mRNA studies, we could not detect expression of TASK-2 protein in the adult mouse retina with the antibody used in this study (Fig. 6D). However, strong TASK-2 immunoreactivity was observed in the conjunctival epithelium which covers the outer surface of the sclera (not included in retina samples used for mRNA analysis) (Fig. 6E,F). Collectively, our data indicate a lack of TASK-2 expression in the adult mouse retina. **TALK-1 and THIK type channels;** Due to lack of detectable mRNA expression in the retina we have not investigated the expression of TALK-1 protein, nor the expression of THIK type channel proteins in the mouse retina.

## Discussion

This study represents the first comprehensive investigation of two-pore domain (K2P) K^+^ ion channel expression within the mammalian retina. Our data indicate that mRNA transcripts for numerous K2P channels are detected in the mouse retina, including inwardly rectifying TWIK-type channels, acid sensitive TASK-type channels, arachidonic acid mechanosensitive TREK-type channels and calcium sensitive TRESK channels, with many K2P channel transcripts showing dynamic patterns of expression throughout the day and during postnatal development. Antibody labelling confirms the widespread expression of K2P channels in the mouse retina and indicates that the highest levels of K2P channel expression are observed in retinal ganglion cells and Müller cells. Overall, levels of K2P channel protein detected are broadly consistent with our mRNA studies in adult tissue and during postnatal development. Typically, channels showing high levels of mRNA expression resulted in high levels of convincing immunoreactivity (TWIK-1, TASK-1, TREK-1, TRAAK), whereas channels showing low levels of mRNA expression typically showed low or absent levels of protein expression (TASK-2). One notable exception was TRESK. Despite high levels of mRNA consistently detected by qPCR analysis (and by gene microarray analysis – Hughes *et al*., unpublished data) we detected only low levels of TRESK protein, with few cells convincingly labelled (including samples collected at various circadian time points). The reasons for this discrepancy are not clear, but likely reflect technical limitations of the antibody used.

There have been a small number of previous reports indicating the expression of certain K2P channels in the mammalian retina, most commonly as part of the initial tissue panel screen conducted following cloning of the specific channel subunits. For example, expression of TRAAK in the mouse retina has previously been confirmed by RT-PCR and *in situ* hybridisation^63^ whereas RT-PCR and immunohistochemistry indicate expression of TWIK-3 (previously termed TWIK-2) in the ganglion cell layer of the mouse retina^64^. However, in both cases the specific cell types expressing these channels has not been determined. A further report has indicated that TASK-2 (a TALK type channel) is expressed in the majority of ganglion cells and both dopaminergic and cholinergic amacrine cells of the rat retina^65^. Our data are largely consistent with these previous reports. In agreement with Fink *et al*.^63^, TRAAK was identified in the mouse retina, although we now confirm this expression is largely restricted to Müller cells with lower levels of expression detected for RGCs. We also confirm that TWIK-3 is expressed within RGCs as previously indicated^64^. However, contrary to previous reports in the rat retina, we did not detect TASK-2 in the adult mouse retina using either qPCR or immunostaining methods. We did however detect TASK-2 mRNA in the postnatal mouse retina, potentially indicating different roles for TASK-2 in the mouse and rat retina. In addition to validating these earlier studies, our data show that the expression of K2P channels in the mammalian retina is highly complex with multiple K2P channels detected within RGCs and Müller cells.

Our data indicate that at least five and possibly as many as eight distinct K2P channels may be expressed within RGCs, including inward rectifying TWIK-type channels, acid sensitive TASK-type channels and arachidonic acid mechanosensitive TREK/TRAAK type channels. Based on levels of immunoreactivity, and the number of positively stained cells, TASK-1 is seemingly expressed at the highest levels, followed by TREK-1, TWIK-2, TWIK-1 and TWIK-3, with TREK-2 and TRAAK detected at only at low levels and TRESK only rarely (and inconsistently) detected. Based on the high number of positively stained cells observed for several K2P channel antibodies, it is highly likely that individual RGCs express multiple K2P channels simultaneously - likely generating complex profiles of K^+^ leak conductances within these cells^36^. Further work will be required to determine the precise functional roles performed by K2P channels within RGCs. However, based on their biophysical properties and known roles in other cell types we might expect K2P channels to make significant contributions to setting resting membrane potential and the regulation of action potential firing, as well as potentially performing roles in signal desensitisation and adaptation^11^,^16^,^66^. In addition to their originally prescribed role as background leak channels it is now becoming clear that the function of many K2P channels is far more complex^16^. K2P channels may contribute directly to action potential waveforms and drive repolarisation of rapidly firing neurones^16^, with TASK-1 and TREK-1 capable of supporting action potential generation in the absence of voltage gated K^+^ channels^67^. It is therefore possible that K2P channels perform a number of diverse roles in RGCs, regulating both the likelihood and also the nature of electrical signalling events.

Based on our data the membrane potential of RGCs, and therefore levels of excitability, are likely sensitive to a wide range of physiological and pharmacological factors to which K2P channels show sensitivities. For example TASK-1 channels are characteristically sensitive to changes in extracellular pH and hypoxia^68^,^69^,^70^, whereas TREK-1 channels (and TREK-2 and TRAAK) are mechanosensitive, temperature sensitive, and are directly modulated by lysophospholipids and polyunsaturated fatty acids (PUFAs)^27^,^46^,^71^,^72^. By contrast TWIK channels typically show lower levels of modulation compared to other K2P channels and can be considered as more typical K^+^ leak channels^16^. It would therefore seem that K2P channels are well placed to act as central regulators of RGC excitability (and therefore photosensitivity) under a wide range of conditions. We propose that the differential profile of K2P channel expression likely contributes to the functional diversity of RGC subtypes^7^, and offers a potential mechanism to fine tune the functional properties of individual RGCs under specific physiological conditions. Further work will be required to determine if the differential expression of K2P channels contribute to the different levels of sustained and transient activity observed from distinct subtypes of RGCs^9^.

It is interesting to note that the majority of K2P channels show an up-regulation of expression in the retina between P10 and P14, at a similar time point to the functional maturation of visual pathways of the mouse retina^73^,^74^. This observation may indicate that K2P channels perform important roles in vision. Glutamate is the key neurotransmitter by which bipolar cells transmit rod and cone derived signals to RGCs^75^,^76^,^77^, and a previous study has described the presence of a glutamate sensitive background leak type K^+^ current in cultured mouse RGCs with properties of a TASK like current, most likely TASK-1 or TASK-3^78^. Inhibition of this TASK-type current by the group I mGluR agonist (*S*)-3,5-dihydroxyphenylglycine (DHPG) results in the depolarisation of retinal ganglion cells and increased levels of action potential firing, suggesting a significant role for TASK type channels in the regulation of RGC excitability by glutamate. Based on the results of our immunostaining data it would seem likely that TASK-1 channels, and not TASK-3 channels, may be responsible for the K^+^ leak current identified by these authors. However, in addition to TASK-1 (and TASK-3), several other K2P channels are known to be modulated by glutamate signalling pathways, including TREK-1 and TREK-2^79^,^80^,^81^. It is therefore likely that K2P channels may mediate complex responses to glutamate within RGCs via multiple mechanisms.

Compared to RGCs, only low levels of K2P channel expression were detected within other retinal neurones, including photoreceptors, horizontal cells, bipolar cells and amacrine cells. This pattern of expression is potentially in keeping the nature of electrical activity observed within the retina. With the exception of RGCs (and a few specialised subtypes of bipolar cells^82^ and amacrine cells^83^,^84^), the majority of retinal cell types do not classically encode light signals as patterns of action potential firing but instead show graded changes in membrane potential^2^,^85^,^86^. It is therefore possible that these cell types show less functional reliance on K^+^ leak currents than typical spike firing neurones, such as RGCs. Our data do however indicate the expression of at least four distinct K2P channels within Müller cells, including TWIK-1, TASK-3, TRAAK, and TREK-2. A previous study has reported the presence of a pH sensitive background leak K^+^ current in Müller cells of mice (and also rat and guinea pig)^87^, and expression of multiple K2P channels have been reported in Müller cells of the amphibian retina (*Rana pipiens*) where they have been shown to perform roles in cell volume regulation and responses to retinal ischemia^87^,^88^. Based on our data it is possible that TWIK-1, TASK-3, TRAAK and TREK-2 may perform similar roles in Müller cells of the mammalian retina, and that TASK-3 may be responsible for the pH sensitive current previously described.

In summary we have shown that K2P channels are widely expressed within the adult mammalian retina, with the highest levels of expression detected within RGCs and Müller cells. Our data suggest that K2P channels likely perform important roles in retinal function and vision, and likely contribute significantly to the resting membrane potential and cellular excitability of retinal ganglion cells under a diverse array of physiological conditions. The prominent role of K2P channels in neuroprotective pathways^45^,^46^,^56^,^89^ offers new potential avenues of research into the treatment of retinal disease. Notably, K2P channels may represent valuable targets for manipulation of resting membrane potential and suppression of pathological increases in spontaneous spike firing rates observed from RGCs following retinal degeneration^90^,^91^,^92^.

## Methods

### Animals

All animal procedures were performed in accordance with the United Kingdom Animals (Scientific Procedures) Act of 1986 and the University of Oxford Policy on the Use of Animals in Scientific Research. All experiments were approved by the University of Oxford Animal Welfare and Ethical Review Board, and were conducted under PPL 30/3068.

C3H mice (C3H/He; not carrying *rd* mutation)^93^ were housed under a 12:12 LD cycle with food and water *ad libitum*. For initial qPCR and antibody analysis eyes (n = 5, P130–135) were collected at Zeitgeber time ZT8 (8 hours after light onset) and processed as described below. Developmental and circadian samples (mRNA and retina sections) were originally collected as part of a previous study^94^. Developmental samples were collected from wildtype C3H/He mice at P0, P3, P5, P10, P14 and P30 (n = 6) at ZT8. Circadian samples were collected from wildtype C3H/He mice (>P45) at ZT3, ZT8, ZT13, ZT18 and ZT23 (n = 6).

### qPCR

Following enucleation, retinae were dissected, flash frozen on dry ice and stored at −80 °C prior to use. Tissue was homogenised in TRIzol Reagent (Life Technologies) and total RNA isolated using RNeasy spin columns (Qiagen) with on column DNase treatment (Qiagen). 1 μg of total RNA was reverse transcribed using SuperScript III with oligo(dT)_20_ primers (Life Technologies) and quantitative PCR performed using Quantifast Sybr Green PCR mastermix (Qiagen) on a StepOne thermal cycler (Applied Biosystems) as described previously^95^. Quantification of transcript levels was performed using a comparative CT approach with levels of target gene expression normalised to the geometric mean expression of three house-keeping genes^96^. K2P channel primers were designed using PrimerBlast (NCBI). Primer sequences are shown in Table 2.

### Immunohistochemistry

Preparation and labelling of retina cryostat sections was performed as described previously^97^,^98^. Briefly, 18 μm sections were permeabilised with 0.2% Triton-X100 (Sigma) for 20 minutes, blocked with 10% normal donkey serum (Sigma) before incubation with primary antibodies for 24 h at 4 °C. Details of all antibodies used are shown in Table 3. Secondary antibodies were donkey anti-rabbit, donkey anti-goat and donkey anti-mouse labelled with Alexa488 and Alexa568 fluorophores (Life Technologies) incubated at 1:200 for 2 h at RT. All antibodies were diluted in PBS containing 2% normal donkey serum and 0.2% Triton-X100. Sections were mounted with Prolong Gold anti-fade media containing DAPI (Life Technologies).

### Image collection

Fluorescence images were collected using an inverted LSM 710 laser scanning confocal microscope (Zeiss) and Zen 2009 image acquisition software (Zeiss). Individual channels were collected sequentially. Laser lines for excitation were 405 nm, 488 nm and 561 nm, with emissions collected between 440–480, 505–550 and 580–625 nm for blue, green and red fluorescence respectively. Typically, images were collected using a x40 objective with images collected every 1 μm in the z-axis. Global enhancement of brightness and contrast was performed using ZenLite 2011 software (Zeiss).

### Co-expression analysis

The percentage of retinal ganglion cells (RGCs) showing K2P channel immunoreactivity was determined by manual counting of adult retinal sections co-stained for the RGC marker Brn3a (brain-specific homeobox/POU domain protein 3A) and relevant K2P channel antibodies. Data values shown represent pooled analysis from counting cells from randomly chosen areas of multiple sections (>10) collected from n = 3 mice at P30.

## Additional Information

**How to cite this article:** Hughes, S. *et al*. Expression and localisation of two-pore domain (K2P) background leak potassium ion channels in the mouse retina. *Sci. Rep.* **7**, 46085; doi: 10.1038/srep46085 (2017).

**Publisher's note:** Springer Nature remains neutral with regard to jurisdictional claims in published maps and institutional affiliations.

## Supplementary Material

Supplementary Material

## Figures and Tables

**Table 1:** Summary of K2P channel immunoreactivity in the adult mouse retina.

K2P channel	PR/ONL	INL	RGCs	Müller cells
TWIK-1	−	−	++	++
TWIK-2	−	−	++	−
TWIK-3	−	−	++	−
TASK-1	−	+	+++	−
TASK-3	−	+	−	+++
TREK-1	+	++	+++	−
TREK-2	−	+	+	+++
TRAAK	−	+	+	+++
TRESK	−	−	+/−	−
TASK-2	−	−	−	−

PR; photoreceptors, ONL; outer nuclear layer, RGCs; retinal ganglion cells.

**Table 2:** Primer sequences for qPCR analysis.

Target	Forward Primer 5′–3′	Reverse Primer 3′–5′	Genebank Acc.
KCNK1	CTACCTGGTGTTCGGCGCCG	CTGCGGGTGACATGCACGGT	NM_008430.2
KCNK2v1	ATGCTGCATGCCTCATGCTTGCC	ACACCGTGGCTCCGATGATCA	NM_001159850.1
KCNK2v2	CTGCAGTGATCACCCCCTCGC	TATTCCAAGACGGGCTGCGCG	NM_010607.3
KCNK3	TGGCTCTCATCGTGTGCACCTTC	GAACACCTTGCCTCCGTCCGT	NM_010608
KCNK4	GTGACCCAGCGAACTGGGCC	GCCACGCTCACTCTGCGTGT	NM_008431
KCNK5	ACCCATGGCTGAGGCACCCT	AGGCCTGTCCTGGGCCAACA	NM_021542
KCNK6	TCCCAGACTCAGTGCCATGCTATGG	TGTGCTCTCAGGACCGCACCTA	NM_001033525
KCNK7	TTGCTGCCTGCCCATGGACG	CTCCCGCTCAGGTCCCCCAA	NM_010609
KCNK9	AGCGGCAGAACGTGCGTACC	AGGTGTTCATGCGCTCGCCC	NM_001033876
KCNK10	GCGAGACCCAACCACTCCGC	GGCCCGGAAGACAAGGCCAC	NM_029911
KCNK12	CACGCTGCGCAACTTCAGCG	GCGCAGCCGAACAGTCCGTA	NM_199251
KCNK13v1	CTCCAGAGCTCATGCGGTCCG	TGCTTCGACTTGGTGCGAGGG	NM_001164427.1
KCNK13v2	GGTGCGTTTGGGAAGCGGTCC	GGTCCGGGTTCCTCGGAGCAT	NM_001164426.1
KCNK13v3	ACCTGTTCCACAGAGCAGCGGTC	TCGGTGCCTCTCCAGGTGGATC	NM_146037.2
KCNK15	TACCGCTTCTCCGCCGACGA	AGGCAGCGCTTGGCTGTCAG	NM_001030292.1
KCNK16	AGACCAGCAGGCCCTGGAGC	ATGCCTGGGGTGGTCCTCCC	NM_029006.1
KCNK18	GCTCTGCCGGAAGCAGCCTG	CCTCTCCACCTGCTGGCCCA	NM_207261.3
*GAPDH*	TGCACCACCAACTGCTTAG	GATGCAGGGATGATGTTC	NM_008084.2
*ARBP*	CGACCTGGAAGTCCAACTAC	ATCTGCTGCATCTGCTTG	NM_007475.5
*PSMB2*	AAATGCGGAATGGATATGAAT	GAAGACAGTCAGCCAGGTT	NM_011970.4
*Β-actin*	ACCAACTGGGACGATATGGAGAAGA	CGCACGATTTCCCTCTCAGC	NM_007393.3

v1, v2 and v3 refer to splice variant isoforms of these genes.

**Table 3:** Primary antibodies used for immunostaining.

Target	Host	Source	Dilution
KCNK1 (TWIK-1)	Rabbit	Abcam, ab8397	1:1000
KCNK2 (TREK-1)	Rabbit	Abcam, ab83932	1:1000
KCNK3 (TASK-1)	Rabbit	Abcam, ab83725	1:1000
KCNK4 (TRAAK)	Rabbit	Abcam, ab83726	1:1000
KCNK5 (TASK-2)*	Rabbit	Abcam, ab113966	1:100–3200
KCNK6 (TWIK-2)	Rabbit	Abcam, ab83728	1:500
KCNK7 (TWIK-3)	Goat	Santa Cruz Biotech, sc-241107	1:1000
KCNK9 (TASK-3)	Rabbit	Abcam, ab83742	1:1000
KCNK10 (TREK-2)	Rabbit	Abcam, ab84210	1:1000
KCNK18 (TRESK)	Rabbit	Abcam, ab83930	1:400
Glutamine synthetase	Mouse	Abcam, ab64613	1:1000
Brn3a	Goat	Santa Cruz Biotech, sc-31985	1:1000

^*^This antibody did not produce detectable levels of staining in the mouse retina under any conditions tested.
