# Supplementary material for: Expression and localisation of two-pore domain (K2P) background leak potassium ion channels in the mouse retina
Source: Sci Rep. 2017 Apr 26;7:46085. doi: 10.1038/srep46085 (PMC5405414; doi:10.1038/srep46085)
Supplement: Supplementary Material [file srep46085-s1.pdf]

Supplementary Material

Expression and localisation of two-pore domain (K2P) background leak potassium ion channels in the mouse retina

Steven Hughes*, Russell G. Foster, Stuart N. Peirson and Mark W. Hankins*

The Nuffield Laboratory of Ophthalmology, Sleep and Circadian Neuroscience Institute, Nuffield Department of Clinical Neurosciences, University of Oxford, Sir William Dunn School of Pathology, OMPI G, South Parks Road, Oxford, OX1 3RE, UK

*** Joint corresponding authors**: steven.hughes@ndcn.ox.ac.uk; mark.hankins@eye.ox.ac.uk

Figure S1. K2P channel mRNA expression throughout development and at different times of day.

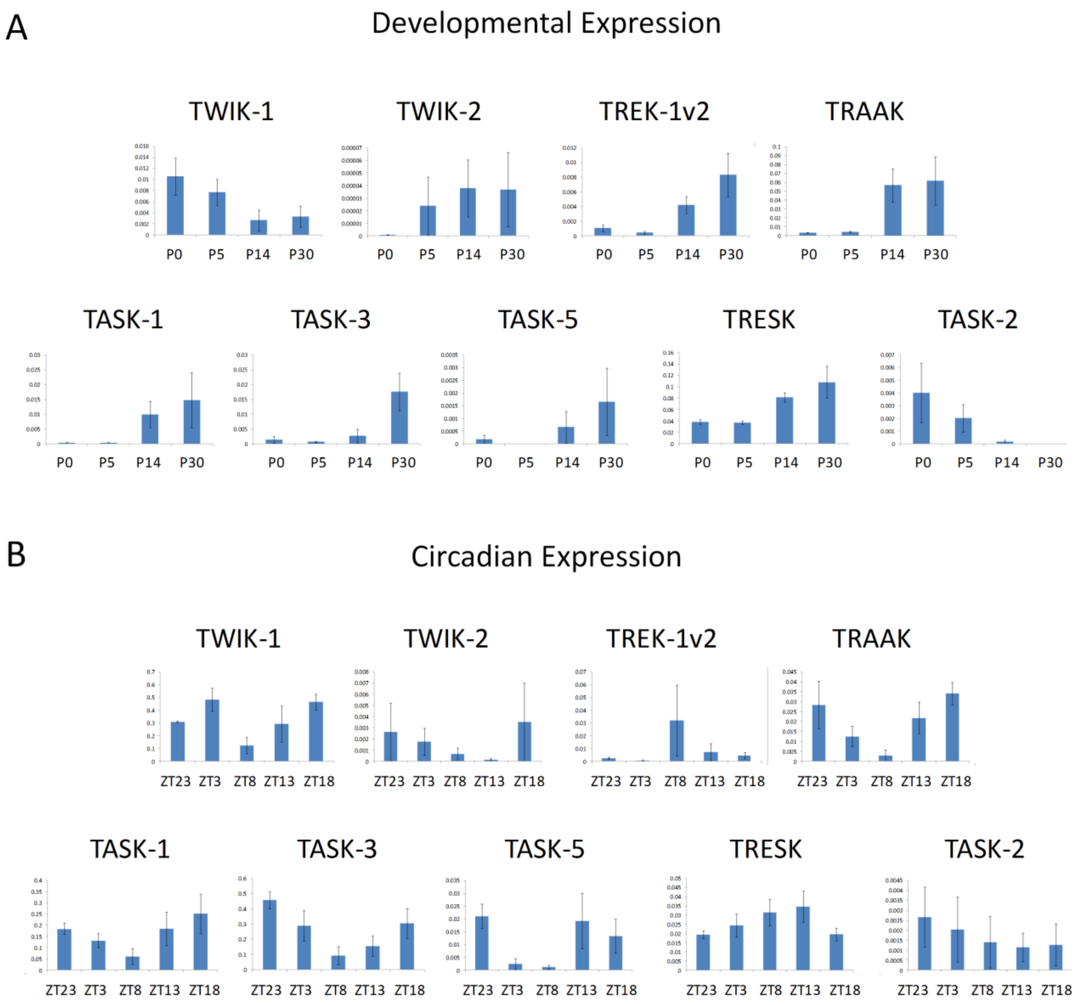

Figure S1. K2P channel mRNA expression throughout development and at different times of day. A) qPCR analysis of K2P channel expression in mouse retina at different developmental time points (n=5, tissue collected at Zeitgeber time ZT8, 8 hours after light onset). B) qPCR analysis of K2P channel expression in mouse retina at different times of day (n=4, mice housed under 12:12 light dark cycles). Data are shown normalised to the geometric mean expression of three housekeeping genes.

Figure S2. Expression of the weak inward rectifying TWIK-1 K2P channel throughout postnatal development of the mouse retina

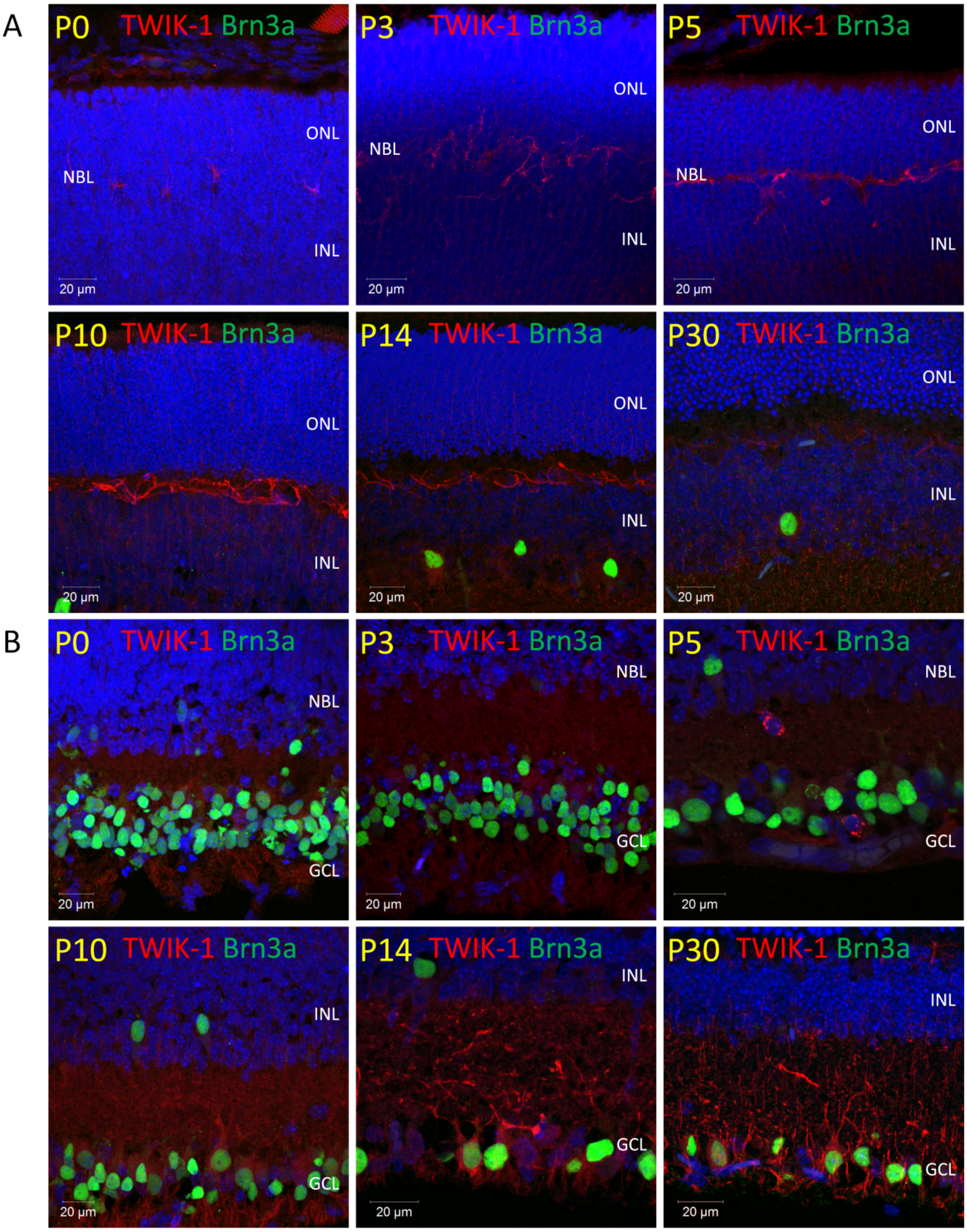

Figure S2. Expression of the weak inward rectifying TWIK-1 K2P channel throughout postnatal development of the mouse retina. A) Images showing levels of TWIK-1

immunoreactivity detected in horizontal type cells of the neuroblastic cell layer and forming outer and inner nuclear layers at P0.P3, P5, P10, P14 and P30. Note the lack of TWIK-1 immunoreactivity in the outer nuclear layer at P30. B) Images showing levels of TWIK-1 immunoreactivity detected in Brn3a positive RGCs of the forming ganglion cell layer at P0.P3, P5, P10, P14 and P30. Note the upregulation of TWIK-1 immunoreactivity at P14 and P30. NBL; neuroblastic layer. ONL; outer nuclear layer, INL; inner nuclear layer, GCL; ganglion cell layer, Brn3a; brain-specific homeobox/POU domain protein 3A, DAPI nuclear counterstain is shown in blue.

Figure S3. Expression of the weak inward rectifying TWIK-2 K2P channel throughout postnatal development of the mouse retina.

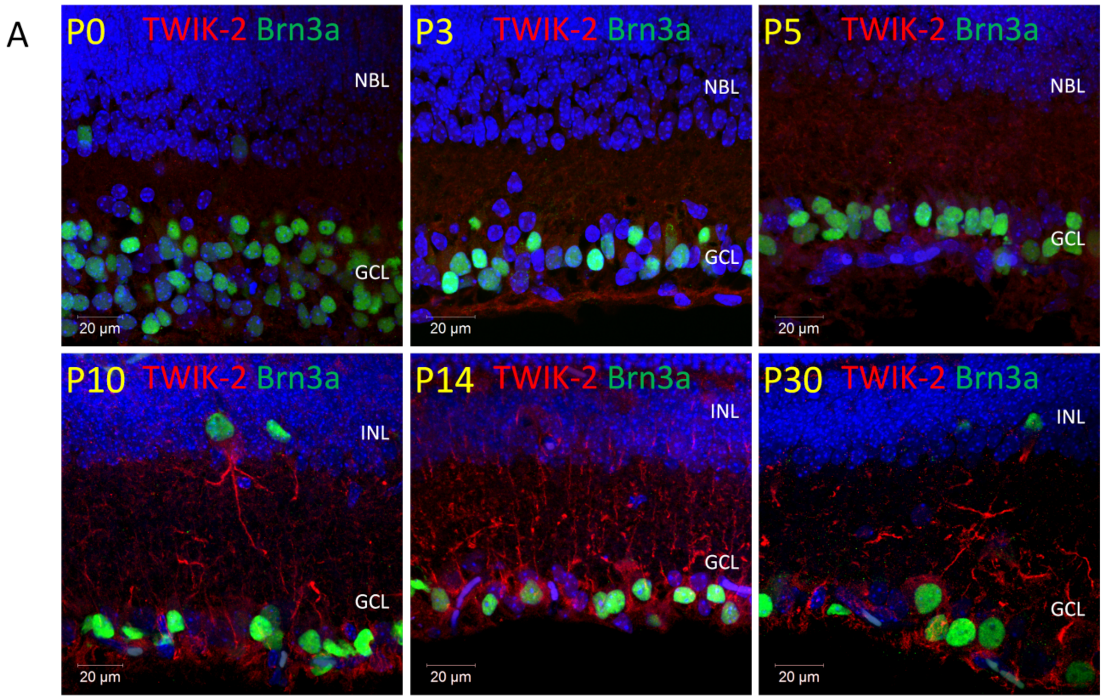

Figure S3. Expression of the weak inward rectifying TWIK-2 K2P channel throughout postnatal development of the mouse retina. A) Images showing levels of TWIK-2 immunoreactivity detected in Brn3a positive RGCs of the forming ganglion cell layer at P0.P3, P5, P10, P14 and P30. Note the upregulation of TWIK-2 immunoreactivity at P10 compared to earlier time points. NBL; neuroblastic layer. ONL; outer nuclear layer, INL; inner nuclear layer,

GCL; ganglion cell layer, Brn3a; brain-specific homeobox/POU domain protein 3A, DAPI nuclear counterstain is shown in blue.

Figure S4. Expression of the acid sensitive TASK-1 K2P channel throughout postnatal development of the mouse retina

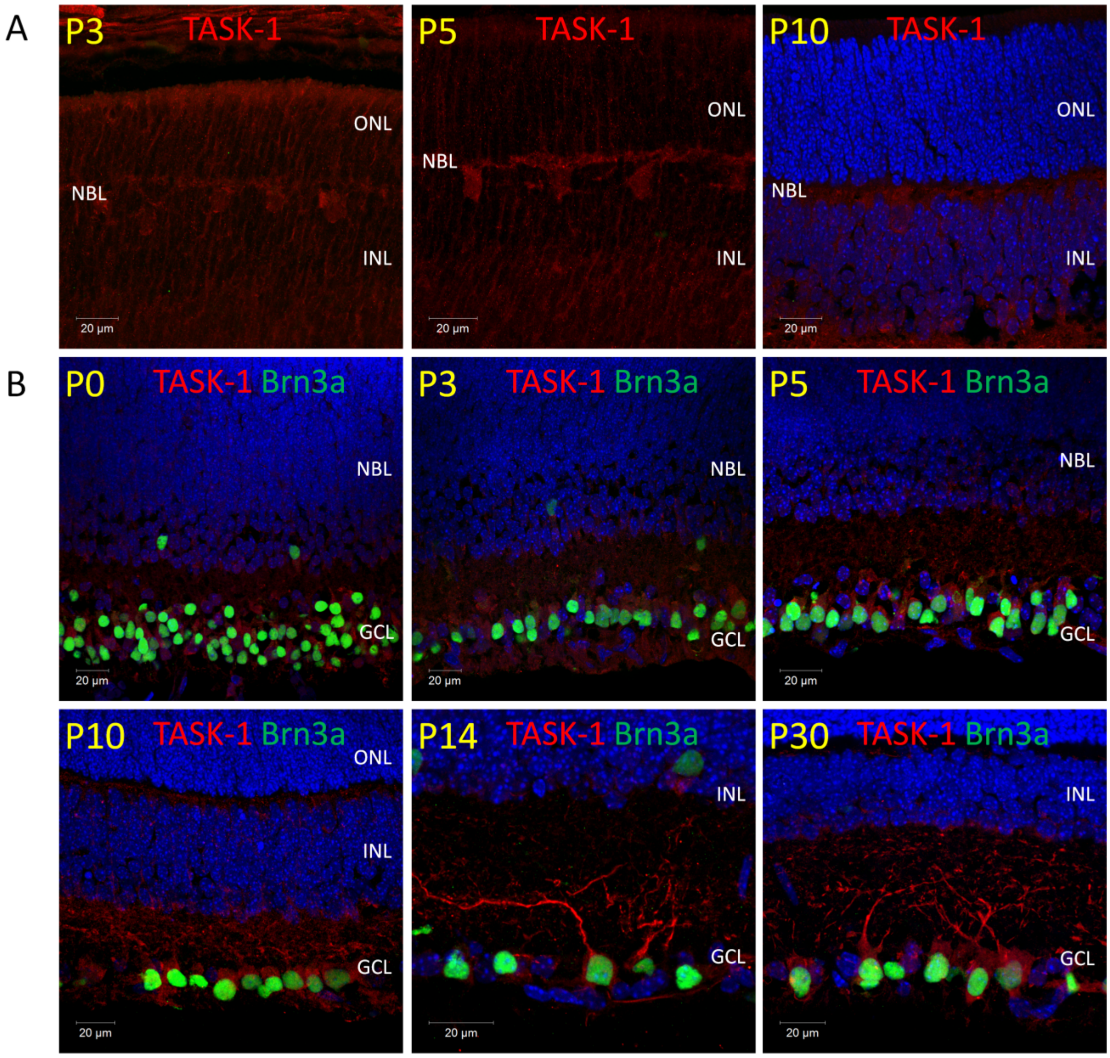

Figure S4. Expression of the acid sensitive TASK-1 K2P channel throughout postnatal development of the mouse retina. A) Images showing levels of TASK-1 immunoreactivity detected in horizontal type cells of the neuroblastic cell layer and forming outer and inner nuclear layers at P3, P5 and P10. TASK-1 immunoreactivity is not detected in the outer nuclear layer post P10. B) Images showing levels of TASK-1 immunoreactivity detected in Brn3a positive RGCs of the forming ganglion cell layer at P0.P3, P5, P10, P14 and P30. TASK-1

immunoreactivity is detected within Brn3a cells as early as P5, with a clear upregulation of detected at P14 and P30. NBL; neuroblastic layer. ONL; outer nuclear layer, INL; inner nuclear layer, GCL; ganglion cell layer, Brn3a; brain-specific homeobox/POU domain protein 3A, DAPI nuclear counterstain is shown in blue.

Figure S5. Expression of the arachidonic acid sensitive mechanosensitive TREK-1 K2P channel throughout postnatal development of the mouse retina

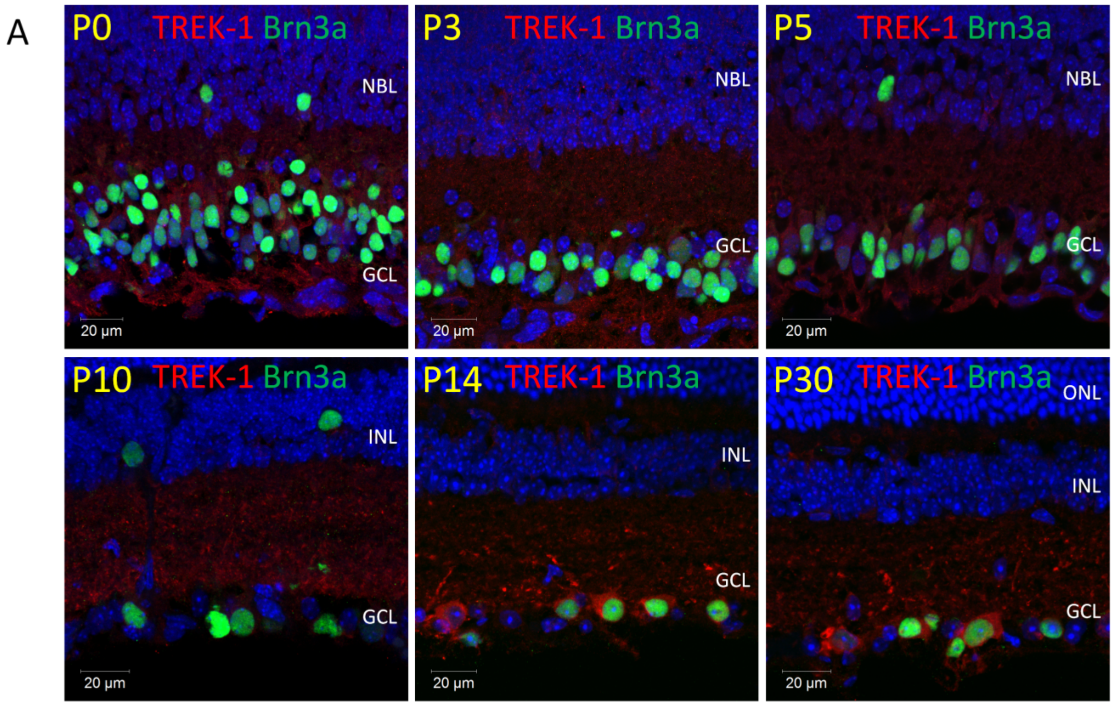

Figure S5. Expression of the arachidonic acid sensitive mechanosensitive TREK-1 K2P channel throughout postnatal development of the mouse retina. A) Images showing levels of TREK-1 immunoreactivity detected in Brn3a positive RGCs of the forming ganglion cell layer at P0.P3, P5, P10, P14 and P30. Note the upregulation of TREK-1 immunoreactivity at P14 and P30. NBL; neuroblastic layer. ONL; outer nuclear layer, INL; inner nuclear layer, GCL; ganglion cell layer, Brn3a; brain-specific homeobox/POU domain protein 3A, DAPI nuclear counterstain is shown in blue.
